# Neurological Manifestations of Primary Immunodeficiencies

**Published:** 2018

**Authors:** Zahra CHAVOSHZADEH, Amir HASHEMITARI, Sepideh DAROUGAR

**Affiliations:** 1 Department of Immunology and Allergy, Mofid’s Children Hospital, Shahid Beheshti University of Medical Sciences, Tehran, Iran; 2 East London NHS Foundation Trust,London,United Kingdom; 3 Pediatric Respiratory Diseases Research Center, National Research Institute of Tuberculosis and Lung Diseases (NRITLD), Shahid Beheshti University of Medical Sciences, Tehran, Iran

**Keywords:** Neurological, Primary immunodeficiencies, Neurologic signs and symptoms

## Abstract

**Objective:**

Primary immunodeficiencies (PID) are a heterogeneous group of disorders with a variable clinical spectrum of manifestations. The central nervous system may be involved in PID with symptoms which may present initially or develop at later stages. The purpose of this study was to review the neurological manifestations of different PID syndromes.

**Materials & Methods:**

We focused on 104 selected studies on PID with certain neurological abnormalities which may accompany these disorders or may later signify a PID in their course.

**Results:**

Diverse neurological deficits accompanying certain PIDs may be mild or they may greatly influence the course of the disease with major impacts on the quality of life of these patients.

**Conclusion:**

Early recognition and treatment is important to prevent or reduce future irreversible neurological sequelae. Therefore physicians should be aware of the neurological features accompanying PID.

## Introduction

Primary immunodeficiencies (PID) are a heterogeneous group of 354 distinct disorders with 344 different gene defects (1) with a variable clinical spectrum of manifestations. A diagnosis of PID will often be considered with a predisposition to frequent, severe or unusual infections, autoimmune disorders and malignancies or allergic disorders (2). 

PIDs have been classified practically according to the affected immune function to the following groups: immunodeficiencies affecting cellular and humoral immunity, combined immunodeficiencies with associated or syndromic features, antibody deficiencies, diseases of immune dysregulation, congenital defects of phagocytes, defects in intrinsic and innate immunity, autoinflammatory disorders, complement deficiencies and phenocopies of PIDs (3). The central nervous system may be involved in PIDs with symptoms which may present initially or develop at later stages. The neurological symptoms may vary from mild cognitive defects to severe disabilities (4). Physical examination may give the clinician valuable clues to the cause of PIDs that underlie the neurological signs. Certain neurological abnormalities may later signify a PID. Therefore physicians should be aware of the neurological features accompanying immunodeficiencies. Neuromascular abnormality presenting with ataxia may be the first indicator of ataxia-telengiectasia. Flaccid paralysis after live poliovirus immunization may suggest combined immunodeficiency or antibody defects. Pernicious anaemia may later result in neurological deficits in untreated CVID patients. Cognitive impairment, nystagmus and cerebellar, spinal and peripheral neuropathies are neurologic features seen in Chediac-Higashi Syndrome. Patients with Griscelli syndrome may present with seizures, ataxia and occulomotor and reflex abnormalities. DiGeorge anomaly may present with subtle developmental delays later manifesting as problem with school performances (5). Early recognition and treatment may prevent or reduce future irreversible neurological sequelae (4). 

Neurological manifestations will be discussed in detail according to the specific classification of primary immunodeficiencies to assist the treating physicians’ timely diagnosis and prompt treatment in order to avoid irreversible neurologic sequelae. The categorization of the inborn errors of immunity is based on the International Union of Immunological Societies: 2017 Primary Immunodeficiency Diseases Committee Report on Inborn Errors of Immunity (1).

## Materials & Methods

The last version of IUIS Primary Immunodeficiencies Committee Report (1) on Inborn Errors of Immunity was reviewed first to select certain PIDs with neurological manifestations. Then a review of literature was started according to specific PID associated with neurological manifestations with a focus on 104 selected studies.

## Discussion

Immunodeficiencies affecting cellular and humoral immunity

 Severe combined immunodeficiencies defined by CD3 T cell lymphopenia

**Adenosine Deaminase deficiency (ADA)**

ADA is a ubiquitous enzyme in purine salvage pathway which is also expressed in both the peripheral and central nervous systems (6). ADA deficiency is caused by mutations in the ADA gene and is known as one of the most prevalent forms of severe combined immunodeficiencies (6). The most common manifestations include recurrent and opportunistic fungal, viral and bacterial infections, lymphopenia and failure to thrive (7). The main neurologic manifestations of these diseases result from accumulation of adenosine metabolites in basal ganglia and thalamus which are rich in adenosine receptors. These neurologic abnormalities include motor delay, hypotonia, mental retardation, learning disability, hyperactivity, attention deficit, behavioural abnormalities, reduced verbal expression, seizure and sensorineural deafness (4, 8). One infant has been reported with nystagmus and difficulty in focusing gaze was found to have brain atrophy on MRI (9).

**DNA Ligase IV deficiency**

This autosomal recessive form of SCID is caused by an impairment of the DNA damage repair process with a pronounced radiosensitivity (10). DNA double-strand break repair via non-homologous end-joining (NHEJ) is involved in recombination of immunoglobulin and T-cell receptor genes. Mutations in NHEJ components may lead to microcephaly and immunodeficiency (11).The neurological manifestations of this disease include microcephaly and developmental delay (12). 

**Cernunnos deficiency**

This is another rare autosomal recessive form of SCID with severe T and B lymphopenia and dysgammaglobulinemia in addition to radiosensitivity caused by mutations in the CERNUNNOS or XRCC4-like factor (XLF). Microcephaly and developmental delay are the prominent neurological features (4, 8). 

**CID with associated or syndromic features**

**Ataxia-Telengiectasia**

This autosomal recessive complex disorder with substantial severity in affected individuals (13) is characterized by ataxia, ocular and cutaneous telengiectasia, radiosensitivity, immunodeficiency, increased predisposition to malignancies (14) and elevated serum alpha fetoprotein level. Sinopulmonary infections (15-17) are common in these patients with development of chronic lung disease in 25% (18). The most common immunologic abnormalities are low serum levels of one or more classes of immunoglobulin, failure to make antibodies in response to vaccines and lymphopenia (19). The ataxia telengiectasia mutated (ATM) gene is involved in the response to DNA damage in the nervous system which leads to impaired apoptosis and elimination of the damaged neurons and neurodegeneration (4). Progressive neurodegeneration which mainly occurs with a widespread loss of Purkinje cells in cerebellum is the hallmark of ataxia-telengiectasia, manifesting as head and trunk swaying and gait abnormalities (8, 13). It is followed by basal ganglia dysfunction later in the course of the disease characterised by hypotonia, tremor and choreoathetosis. These patients are usually confined to a wheelchair by the age of 10-12 years of age. In physical examination, further evaluation reveals diminished or absent tendon reflexes, positive Babinski sign, loss of vibration sense, abnormal ocular motility, reduction in sensory conduction velocity and axonal degeneration of peripheral nerves (8).

There are also Ataxia Telengiectasia-Like Disorders (ATLD) which are clinically similar to ataxia telengiectasia although without telangiectasia and a slower progression of neurologic manifestations. These patients remain ambulatory until their third decades of lives (8, 20).

**Nijmegan Breakage Syndrome**

This is a rare autosomal recessive, multisystemic disease of chromosomal instability presenting at birth with microcephaly but no additional neurologic manifestation (21). The mutated gene (NBS) has a critical role in responding to DNA damage. The defect leads to neuronal loss and microcephaly. “Other manifestations include combined cellular and humoral immunodeficiency with recurrent sinopulmonary infections, a strong predisposition to develop malignancies and radiosensitivity” (21). Due to the radiation hypersensitivity in these patients, they are at increased risk of developing brain malignancies such as medulloblastoma (22). These patients may exhibit progressive mental retardation which becomes more evident after age of 14 years (23). As well as brain developmental abnormalities including partial collosal agenesis, hydrocephaly, colpocephaly, neuronal migration abnormalities. The volume of the frontal lobe may be reduced and the gyral pattern may become simplified (24). 

**Immunodeficiency, Centromeric Region Instability and Facial Anomalies Syndrome**

This genetic disease is due to a mutation in DNA methylteransferase gene (25) which causes DNA hypomethylation. It is not identified how these hypomethylated areas of the genome result in the features of this syndrome but it appears that the expression pattern of the genes essential for a functional immune system, craniofacial development and neurogenesis are altered in the deficient cells (26). ICF patients have severe respiratory infections and recurrent gastrointestinal infections due to immune abnormalities which include hypogammaglobulinemia despite normal B cell counts and a mild reduction in immune response. Reduced T cell counts have been detected in approximately half of the cases (99). Affected patients exhibit mental retardation, hypotonia and ataxia in addition to above immune abnormalities (25, 27).

**Riddle Syndrome**

This syndrome is characterized by a defect in DNA damage response causing immunodeficiency and increased radiosensitivity accompanied by neurologic symptoms and growth delay due to an increased apoptosis and reduced proliferative capacity during brain development presenting as mild motor control and learning difficulties (28). 

**DiGeorge Syndrome**

In addition to the characterizing triad of cardiac defects, hypocalcemia and cellular immune deficiency, there may be neurological manifestations which present with developmental delay in motor, language and speech areas, neuropsychiatric problems and epilepsy (4). Most patients show a hemizygous chromosome 22q11.2 deletion, and most of them with DiGeorge syndrome show mild to moderate T cell lymphopenia. The most common neurologic manifestations include cognitive, neurologic and psychiatric abnormalities (8). Nearly 50% percent of school-age patients have problems with persistent language difficulties while their full-scale IQ scores are within normal to moderately retarded range with math scales lower than reading and spelling (29, 30). Behavioural abnormalities include anxiety disorders, schizophrenia, ADHD, mood disorders and flat effect in childhood (31) and schizophrenia and bipolar disorders later during adulthood. Neural tube defects, polymicrogyria, volume reduction in the corpus callosum, amygdalae and temporoparietal regions (29, 32) and myelomeningocele (33) are structural malformations which have been described in these patients.

**Hyper-immunoglobulin E syndrome**

These syndromes are characterized by recurrent skin and pulmonary infections in the presence of elevated IgE concentrations and usually eosinophilia. The hyper-IgE syndromes have four distinct genetic causes including mutations in signal transducer and activator of transcription 3 (STAT3), dedicator of cytokinesis 8 (DOCK8), tyrosine kinase2 (TYK2) and phosphoglucomutase 3 (PGM3) (1, 34). Vascular abnormalities, impaired myelination or autoimmunity are the main suggested causes for neurologic manifestations in these patients (35, 36). Intracranial manifestations including Chiari I malformations and focal hyperintensities on brain MRI are the two findings in AD-HIED which are typically asymptomatic (37). Other CNS reported abnormalities include vasculitis resulting a right parietal infarction and thrombosis of the left posterior inferior cerebellar artery (38, 39). In DOCK8 deficiency neurologic manifestations include vasculitis, ischemic infarction, hemiplegia, facial paralysis, subarachnoid hemorrhages and progressive multifocal leukoencephalopathy (34, 40). Patients with PGM3 mutations are reported to carry a high risk of early onset neurological impairment including developmental delay, ataxia, dysarthria, psychomotor retardation, hypotonia, sensorineural hearing loss, seizures and myoclonus (34, 41).

**Schimke Immuno-osseous Dysplasia**

This autosomal recessive disorder is caused by mutation in the SMARCAL1 gene which encodes a chromatin remodeling protein. Defective cellular immunity causes recurrent bacterial, fungal and viral infections which in association with short stature and progressive renal insufficiency characterizes the disorder (4). Neurological manifestations include early-onset cerebral ischemic attacks, Migraine-type headaches (42), optic neuropathy, seizures, mental retardation and behavioural changes (4, 43). Progressive aterosclerosis and intrinsic neurovascular defects are considered as the underlying pathologic involvement causing the above manifestations (44).

**Hoyeraal-Hreidarsson syndrome**

This is an X-linked recessive disorder of telomere dysfunction which represents a severe form of dyskeratosis congenita characterized by a classic triad of reticular skin pigmentation, dysplastic nails and oral leukoplakia (45). Pancytopenia and severe combined immunodeficiency (characterized by a decreased B cells count and Natural Killer cells and low immunoglobulin levels that can affect all immunoglobulin subtypes) may eventually develop (46). Dyskerin, a highly conserved protein is the product of DKC1 gene, involved in ribosome biogenesis and acts as a component of telomerase complex (8). Since proper telomerase activity is essential for early development of neural progenitor cells and neurons, its defective function may lead to increased susceptibility to DNA damage and apoptosis and the resultant neurologic deficits. These neurological abnormalities include microcephaly, psychomotor retardation, spasticity, cerebellar hypoplasia, ataxia (8), seizures and axial hypotonia (47).

**Purine Nucleoside Phosphorylase Deficiency**

This rare autosomal recessive metabolic disorder, similar to ADA deficiency, results from impaired purine salvage pathway which causes combined immunodeficiency in association with prominent neurological abnormalities (48). “Mutations in PNP results in impaired function of enzyme leading to accumulation of purine metabolites such as deoxy-guanosine triphosphate (dGTP) which preferentially is accumulated in mitochondria and will affect adversely mitochondrial DNA repair and therefore may induce subsequent neuronal cell apoptosis” (8). Patients present with T-lymphocytopenia (49). Infectious complications include recurrent upper and lower respiratory tract infections and otitis media (48, 49). Neurological findings found in more than half of the patients often precede infections and autoimmunity (4) and include motor system dysfunction, cerebral palsy, hyper/ hypotonia, spastic paresis, tremor, ataxia, mental retardation, developmental delay, behavioural problems, cerebrovascular accidents and sensorineural deafness (4, 8, 48, 50). 

**Hepatic Veno-Occlusive Disease with Immunodeficiency**

This is caused by homozygous mutation in the autosomal SP110 gene. The onset of clinical features usually occurs before the age of six months with hepatic sinusoidal obstruction. Serum Ig levels are low. T cell subsets and B-cell numbers are reduced. Neurological abnormalities include leukodystrophy and extensive cerebral necrosis on postmortem examination are found in one third of the patients (51).

**VICI Syndrome (Immunodeficiency with Cleft Lip/Palate, Cataract, Hypopigmentation and Absent Corpus Collasum)**

This rare autosomal recessive disorder is caused by mutation in the EPG5 gene which results in defective autophagy (52). Clinical findings include cataracts, cleft lip and palate, progressive cardiomyopathy, variable pigmentary defects and combined immunodeficiencies. Neurologic manifestations include microcephaly, seizures, nystagmus, callosal agenesis, hypotonia and severe psychomotor and growth retardation (53). Some patients show more structural CNS abnormalities (52, 53). 

**Predominantly Antibody deficiency**

Common variable immunodeficiencies and agammaglobulinemia (x-linked or autosomal recessive) are the most frequent forms of presentations of antibody deficiency. Symptoms mainly consist of respiratory or gastrointestinal involvement. However, neurologic manifestations may also occur. 

**Common Variable Immunodeficiency**

Neurologic manifestations are a complex of diverse and rare complications of CVID including brain or spinal cord infections (meningitis as the most common neurologic manifestation), autoimmune involvements (featuring as axonal sensorimotor polyneuropathy) (54), transverse myelitis (55), progressive neurodegeneration (56), cerebral vasculitis (causing recurrent occipital headaches), brain granulomatous lesions (57), free radical mediated neuronal damage due to vitamin E deficiency (including sensory loss, ataxia, retinitis pigmentosa) (58, 59), subacute combined degeneration of the cord secondary to vitamin B12 deficiency, peroneal muscular atrophy, Guillain-Barre syndrome and myasthenia gravis (55).

**Agammaglobulinemia**

In patients with agammaglobulinemia symptoms mainly consist of recurrent respiratory or gastrointestinal infections. Serum immunoglobulins are severely decreased and usually undetectable (60). CNS complications may arise from persistent enteroviral infections which may lead to fatal encephalitis or dermatomyositis-meningoencephalitis syndrome (61). “Also, a progressive encephalopathy of unknown etiology has been described in patients with x-linked agammaglobulinemia with a uniform clinical course including cognitive and movement disorders” (62).

**Disorders of Immune Regulation**

**Chediac Higashi Syndrome**

Patients with this autosomal recessive lethal disorder present with recurrent respiratory and bacterial skin infections due to severe immunodeficiency, partial oculocutaneous albinism, bleeding tendency and progressive neurological features (63). This syndrome is caused by mutations in the lysosomal trafficking regulator gene (LYST) (4, 8). Neurologic abnormalities include weakness, areflexia, sensory deficits, peripheral polyneuropathy, progressive neurodegeneration, cognitive defects, dementia, ataxia, tremor, seizures, autonomic dysfunction and parkinsonian syndrome (64). Unfortunately the progression of neurological manifestations is not arrested by HSCT (64, 65).

**Hermansky-Pudlak syndrome type 2**

Among Hermansky Pudlak syndromes, this type is characterized by an immunodeficiency due to neutropenia and T lymphocyte dysfunction which differentiates it from other types of this syndrome (66). These patients usually suffer from recurrent bacterial infections, particularly respiratory illnesses, hepatosplenomegaly, lung fibrosis, dysmorphia, oculocutaneous albinism, thrombocytopenia and neurological abnormalities (67). Neurological manifestations include developmental delay, generalized seizures, microcephaly, hearing impairment and intention tremor (68, 69).

**Familial Hemophagocytic Lymphohistiocytosis**

FHL comprises a group of autosomal recessive life-threatening disorders characterized by uncontrolled proliferation of activated lymphocytes and macrophages associated with massive secretion of cytokines infiltrating into organs including lymph nodes, bone marrow, liver, spleen and central nervous system (70, 71). CNS infiltration begins in the meninges, progressing to diffuse infiltration of the white matter followed by necrosis and focal demyelination (4, 71). Therefore neurological manifestations vary depend on the areas involved including seizures, decreased level of consciousness, facial palsy, irritability, bulging fontanel, dysphagia, dysarthria, CSF abnormalities (leukocytosis), ataxia, nystagmus, visual disturbance, neck stiffness, cranial nerve palsies and brain death (72-74).

**Congenital defects of phagocytic number or function**

**Chronic Granulomatous Disease**

Chronic granulomatous disease is the most common inherited disorder of phagocytic cells. It results from an inability of phagocytes to produce bactericidal superoxide anions due to defects of the NADPH oxidase system and it leads to the defect in killing of a specific spectrum of bacteria and fungi resulting in concomitant hyper-inflammation and tissue granuloma formation (75). Neurological manifestations are reported due to brain abscesses which are rare events in CGD (76). Other complications include white matter disease, CNS granulomatous disease and leptomeningeal and focal brain infiltration by pigmented lipid-laden macrophages (75).

**Severe Congenital Neutropenia**

This term is used for a heterogeneous group of primary immunodeficiencies including sporadic autosomal recessive, autosomal dominant and x-linked types (39) of which the autosomal recessive type with a homozygous mutation in antiapoptotic protein, HAX1, accounts for one third of these patients and has been identified as the only subtype associated with neurologic abnormalities (40). Neurologic manifestations include developmental delay, cognitive defects, speech defects, attention deficit, hyperactivity, learning disabilities and epilepsy (77-79). 

**Schwachman-Diamond Syndrome**

This is a multisystem disorder characterized by bone marrow failure, exocrine pancreatic insufficiency and metaphyseal chondrodysplasia. These features are attributed to mutation in SBDS gene (80). However the precise pathologic pathways with the potential ability to disrupt the protein’s function remain to be identified. Neurological findings include neuropsychological abnormalities. These patients not only suffer from behavioural problems but also experience developmental retardation, low intelligence, cognitive deficits, hypotonia and visual motor dysfunction (81). Brain structural anomalies including callosal agenesis, delayed myelination and decreased gray and white matter volumes have been detected (82).

**Leukocyte Adhesion Deficiency type 2**

LAD syndromes consist of rare autosomal recessive disorders among which only LAD type 2 presents with neurologic abnormalities. These patients are characterized by recurrent bacterial infections without pus formation in the first years of life and severe mental retardation later (83). Other neurologic manifestations include autistic features, microcephaly, muscular hypotonia and cerebral atrophy (83, 84). Strong delays in directional movement and severe retardation in speech development have been also reported (84). Impaired fucose metabolism has been suggested as the cause of neurologic manifestations (85).

**β-Actin deficiency**

This autosomal dominant immunodeficiency disorder caused by a non-lethal mutation, characterized by defects in neutrophil migration, mental retardation, short stature, thrombocytopenia and photosensitivity (86). The hallmark of the disorder is recurrent bacterial and fungal infections without pus formation. The neurologic manifestations of this defect are due to the contribution of β-actin in neuronal migration and development and particularly includes mental retardation. 

**Autoinflammatory Disorders**

**Mevalonate Kinase Deficiency (MKD)**

This is a term used for a wide clinical spectrum of autosomal recessive disorders due to a congenital error in cholesterol biosynthesis, with hyperimmunoglobulinemia D syndrome as the benign variant of the disorder and Mevalonic aciduria as the more severe form of MKD (3). Mevalonate kinase is an important enzyme in the biosynthesis of cholesterol. Recurrent, non-infectious febrile episodes lasting 4 to 6 days, usually accompanied by cervical or abdominal lymphadenopathies, splenomegaly, vomiting, diarrhoea, arthralgia and erythematous/ urticarial rash are characteristic. Although the pathogenic mechanisms causing the neurological defects are not understood, the role of cholesterol in brain development and neuronal function cannot be ignored. The neurological manifestations in a subset of patients with hyperimmunoglobulinemia D syndrome include mental retardation, ataxia and epilepsy (87). In patients with mevalonic aciduria due to complete MVK deficiency, neurological manifestations may include dysmorphic craniofacial features, psychomotor retardation, developmental delay, hypotonia and progressive cerebellar ataxia (88, 89). 

**Neonatal Onset Multisystem Inflammatory Disorder**

The disease results from mutations in the CIAS-1 gene which encodes cryopyrin (90). Cryopyrin in association with other proteins form a protein complex called the inflammasome which activates IL-1β to its proinflammatory form. These mutations cause which aberrant activation of the inflammasome (91) are supposed to be responsible for the persistent upregulation of this inflammatory pathway (92). Therefore like other involved organs, the increased IL-1β activity (93) in the brain seems to cause the neurological symptoms which are the prominent manifestations of this disorder. These abnormalities include chronic aseptic meningitis which may cause increased intracranial pressure resulting in hydrocephalus, papilledema and optic nerve atrophy, spastic diplegia, hypotonia, transient episodes of hemiplegia, mental retardation, developmental delay, brain atrophy, headache, early morning nausea, vomiting, progressive sensorineural hearing loss and seizures (94). 

**Blau syndrome**

This rare autosomal dominant syndrome clinically resembles early onset sarcoidosis characterized by granulomatous inflammation mostly involving joints, eyes and skin (95-97) and is caused by mutation in pattern recognition receptor NOD2 gene encoding a protein known as an intracellular sensor for bacterial products (98, 99). This protein is activated after receiving signals from bacterial components resulting in activation of NFkB pathway which regulates innate responses (99). Neurological manifestations are rare and include cranial neuropathies (hearing loss) and transient 6^th^ nerve palsy, cerebral vasculitis and cerebral infarction (100, 101).

Aicardi-Goutieres syndrome

This is a genetically determined encephalopathy caused by mutations in TREX1 genes encoding RNAs involved in removing RNA, leading to the accumulation of endogenous RNA, triggering Toll-like receptor-dependent interferon-α production in the brain with the resultant activation of neurotoxic lymphocytes and immune system in addition to the inhibition of angiogenesis (102, 103). Neurological manifestations include early infantile onset of irritability, spasticity, dystonic posturing, psychomotor retardation and microcephaly, resulting in death in early childhood (104).

**In conclusion, **neurological manifestations of primary immunodeficiencies are common with diverse pathologic mechanisms. Neurological deficits may be mild or they may greatly influence the course of the disease with major impacts on the quality of life of the patients. Some of these complications may even cause death. Neurological manifestations may have an early onset, beginning at birth or they may appear later which in both situations may lead to misdiagnosis and delayed therapy. Therefore a high suspicion of an underlying primary immunodeficiency is important in any type of neurological problems occurring with recurrent infections.

## Authors’ Contribution

 All three authors were involved in data collection, and writing the article.

## Conflict of Interest

The authors declare that there is no Conflict of Interest.
